# COVID-19 Study on Scientific Articles in Health Communication: A Science Mapping Analysis in Web of Science

**DOI:** 10.3390/ijerph19031705

**Published:** 2022-02-02

**Authors:** Carlos de las Heras-Pedrosa, Carmen Jambrino-Maldonado, Dolores Rando-Cueto, Patricia P. Iglesias-Sánchez

**Affiliations:** 1 Faculty of Communications Sciences, Universidad de Málaga, 29016 Málaga, Spain; cheras@uma.es (C.d.l.H.-P.); lrandocueto@uma.es (D.R.-C.); 2 Faculty of Commerce and Management, Universidad de Málaga, 29016 Málaga, Spain; patricia.iglesias@uma.es

**Keywords:** health communication, COVID-19, coronavirus, bibliometric analysis

## Abstract

The COVID-19 pandemic continues to cause a collapse in the health systems and econo-mies of many countries around the world, after 2 years of struggle and with the number of cases still growing exponentially. Health communication has become as essential and necessary for control of the pandemic as epidemiology. This bibliometric analysis identifies existing contributions, jointly studying health communication and the pandemic in scientific journals indexed. A systematic search of the Web of Science was performed, using keywords related to COVID-19 and health communication. Data extracted included the type of study, journal, number of citations, number of authors, country of publication, and study content. As the number of scientific investigations has grown, it is necessary to delve into the areas in which the most impactful publications have been generated. The results show that the scientific community has been quick to react by generating an extraordinary volume of publications. This review provides a comprehensive mapping of contributions to date, showing how research approaches have evolved in parallel with the pandemic. In 2020, concepts related to mental health, mass communication, misinformation and communication risk were more used. In 2021, vaccination, infodemic, risk perception, social distancing and telemedicine were the most prevalent keywords. By highlighting the main topics, authors, manuscripts and journals since the origin of COVID-19, the authors hope to disseminate information that can help researchers to identify subsisting knowledge gaps and a number of future research opportunities.

## 1. Introduction

Almost 2 years after the onset of the COVID-19 pandemic, the whole world is experiencing uncertainty. The current crisis caused by COVID-19 is taking place in a context that is unprecedented for society. In this globalised world, the transit of people between countries, the development and use of Information and Communication Technologies (ICT) [1,2,3,4,5] and the interconnection between economies or the flow of goods, capital and ideas [6] are fully established. This environment facilitates the transmission of the coronavirus or each of its new variants such as Delta or Omicron. Depending on the country, up to six waves can be counted that have left behind thousands of infected and dead people, and where vaccines are considered by governments to be the great saviours [7]. It is in these situations of uncertainty, insecurity or even disbelief on the part of the population where communication and especially health communication and risk communication become essential tools for managing public health [3,4,8,9].

Thus, the World Health Organisation (WHO) [10] has highlighted communication as one of the biggest challenges and has identified risk communication as one of the core competencies needed to deal with a pandemic. Jong-Wook, ex-director of the WHO, said ‘we have recently learned that communication is as necessary an element of epidemic control as laboratory analysis or epidemiology’ [10].

Health communication is a field of research that emerged in the 1980s and focuses on the influence of communication on health-related aspects of a given population [11,12]. Its main objective is to influence individual and collective decisions that lead to improved health [13].

Effective communication in times of pandemic means that all messages should be shared with stakeholders in an open and transparent communication process. The main objective of this process is to rectify possible divergences between the information generated by those who create the information and those who receive it. In this way, public behaviour can be adjusted to proactively address risk [14,15]. But we must also be aware of the information and disinformation to which the population is subjected. In situations such as the current one, of uncertainty, the consumption and use of information by society increases [16,17,18,19]. In the case of the European Union, the arrival of vaccines against COVID-19 [20] has raised new challenges in terms of communication. Part of the public has been vaccinated, trusting in the recommendations of their governments, the European Union [7] and the WHO [21], but an anti-vaccine trend has arisen that has led many countries to create a COVID-19 passport for access to facilities such as museums, restaurants, etc. The information generated by the media and the governments themselves has sometimes been contradictory [3]. Lack of communication strategy and messages have created negative consequences for many citizens [22] and has even become a source of stress for society.

Health communication plays an important role in the society [23]. The ability to communicate quickly and transparently is essential for the effective management of a public health emergency. In response to the exceptional situation caused by the pandemic, scientific production in health communication related to the coronavirus has increased significantly. One of the main tools for understanding scientific productivity are the bibliometric studies that allow the analysis of the activity and indicators of scientific productivity, such as the impact and visibility of scientific publications, manuscripts or authors, collaboration networks [24] or the analysis of scientific maps [25].

According to Newman [26], the analysis of scientific publications plays a decisive role in the visibility, expansion and consolidation of the results of knowledge transmission. This research aims to map scientific production in journals indexed in the Social Sciences Citation Index (SSCI) and Science Citation Index Expanded (SCI-Expanded) categories of the Web of Science (WoS) in the period 2020–2021.

Although there are many recognised databases, currently the two main databases of bibliographic references and citations of academic publications in the world are WoS and Scopus. The former is owned by Clarivate Analytics and the latter by Elsevier. The publications collected in both databases are of scientific content relevant to research in universities, hospitals and health research services, public and private research organisations, technology centres, science parks, etc.

This research focuses on the analysis of the scientific production of all the publications indexed in the categories outlined above and centred on the keywords ‘coronavirus’, ‘COVID-19’ and ‘health communication’.

This study pursues the following objectives:

1. To find out the volume of articles published on COVID-19 and health communication indexed in the WOS database during the pandemic.
2. To identify the main countries, universities, authors and scientific journals publishing research on health communication during this period.
3. To detect the most relevant approaches, methods and lines of research on COVID-19 and health communication.
4. To visually represent the degree of international scientific collaboration and thus serve as a starting point for future research in the area.

## 2. Materials and Methods

For the bibliometric study, the Web of Science (WoS) database was used as a first source of information, from which scientific articles were selected in journals indexed in the categories of Social Sciences Citation Index (SSCI) and Science Citation Index Expanded (SCI-Expanded) that in the period 2020–2021 have dealt with the subject related to: COVID-19 or coronavirus and ‘health communication’. We also obtained manuscripts that referred to the terms COVID-19 or coronavirus and also to COVID-19 or coronavirus by adding the term ‘health’. These two searches have often served to focus the study.

The inclusion and selection criteria, as well as the indices that were applied in the process of searching for publications in WoS, are summarised in Table 1. The different results obtained depending on each of the different searches carried out are shown, with the research objectives being refined as more specific results were obtained in order to deepen the analysis of the articles on COVID-19 or coronavirus and ‘health communication’ as the object of the present study.

The methodological design as well as the resources employed are based on the systematic review of the scientific literature on bibliometric analysis, as well as the scientometric and visual tools and indicators developed by Chen [27]. In turn, the research follows the approach given by Sánchez-Núñez, De las Heras-Pedrosa and Peláez [28], in their scientific mapping analysis for the computational analysis (of the number, increase and typology of scientific production, according to authors, scientific journals, keywords, institutions) and manual analysis of the information extracted from the research publications to determine the predominant treatment in the scientific literature on health communication in coronavirus, related thematic trends in this field, as well as possible areas of scientific application.

Based on the results obtained in WoS, VOSviewer and CitNetExplorer software were used for the analysis of scientific production. The first tool was used to study bibliometric performance indicators and identify citation patterns (countries/regions, authors, organisations, publications and academic journals). Configuration of the VOSviewer analysis [29] was as follows:

(1) Unit of analysis: Authors, Academic Journals, Organizations, Countries/Regions;
(2) Type of network: Citation analysis;
(3) Cluster network design: Network visualization and density visualization.

Therefore, bibliometric networks are analysed to extract information about co-authorship, co-citation, citation networks and keyword co-occurrence in scientific publications [29,30]. CitNetExplorer was used to visualise and analyse the citation networks of selected scientific articles, highlighting the relationship between groups of publications [31].

## 3. Results

According to the scientific cartography obtained and the scientometric indicators calculated with the computational analysis of the data, the results shown in this section are obtained.

### 3.1. Citation Report

After the first search (Table 1), more than half of the articles on coronavirus found (63.38%) correspond to scientific publications in 2021. When the terms ‘health’ or ‘health communication’ are added to ‘coronavirus’ in 2021, the number of articles identified is 63.70% and 57.63%, respectively.

On the citation report under study (Table 2), a total of 1505 citations were obtained. Logically, this figure differs greatly from year to year. In 2020 the number of citations was 184, while in 2021 this figure already amounted to 1309. The average number of citations per article is 5.74 and the H-index is 20.

### 3.2. Analysis of the Main Journals by Number of Publications

#### 3.2.1. Journals with COVID-19 and Health Publications

As a starting point, the main journals that have published manuscripts on the subject of coronavirus and health were analysed. The International Journal of Environmental Research and Public Health ranks first with 4.84% of the total (Table 3).

Researchers from all over the world in the field of health have chosen these journals which, as can be seen in Table 3, except for the Journal of Medical Virology, are all indexed in the first or second quartile of the Journal Citation Report (JCR) for the year 2020: 10 in JCR Q1 and 9 in JCR Q2. Therefore, we can consider them the best journals in the world in the field of health.

This ranking of the journals with the highest number of published articles has as common characteristics in that they are open access and dedicated to publishing high-quality research in the areas of science or social sciences. Moreover, they are online publications, which means that their papers can be published, read and analysed quickly by the scientific community.

At this time of pandemic where the coronavirus has required the efforts of all researchers in the world to understand its implications in record time in order to safeguard the health of the world’s citizens, it is essential to have journals that are rigorous in their peer reviews but also very agile in their publication.

#### 3.2.2. Journals Focus on COVID-19 and Health Communication

In the case of the search on COVID-19 and health communication, the ranking of the 20 journals with the highest number of contributions on this topic is as shown in Table 4. The aim is to associate the manuscripts with this term in order to detect only those articles that refer solely and exclusively to health communication in COVID-19 times. The term ‘Health Communication’ was searched for in the entire text and not only in keywords or manuscript title, obtaining a total of 262 manuscripts.

The inclusion of communication aspects shows that many of the journals, especially the Journal of Health Communication and Health Communication, are indexed in JCR Social Science (Table 4). Related to the citations, the first position is occupied by Health Communication with 383 citations followed by International Journal of Environmental Research and Public Health (IJERPH) with 202 citations. In the case of the top two journals in the ranking, their titles coincide with the search term of this research. Although this is not the case for IJERPH, this journal is clearly divided into sections where there used to be a section called ‘Health Communication’ clearly identifying ‘the use of communication as a strategy to promote health and prevent disease’ [32]. They have now included the term Informatic in this section. It is now called ‘Health Communication and Informatics’.

### 3.3. Analysis of the Main Authors and Number of Publications

The analysis of authors made it possible to identify the most prolific publications in terms of scientific diffusion. Based on the volume of publications in which the concept of ‘Health Communication’ accompanies the concept of COVID-19 or coronavirus, the authors listed in Table 5 are those who have generated the greatest scientific output in 2020 and 2021. On this occasion, those authors whose production was equal to or greater than three articles were selected.

### 3.4. Analysis of Main Co-Authorships—Countries/Regions and Number of Papers

In the studies by country, the USA stands out, doubling its publications with respect to the rest of the countries and regions of the world, followed by China.

**Figure 1:**
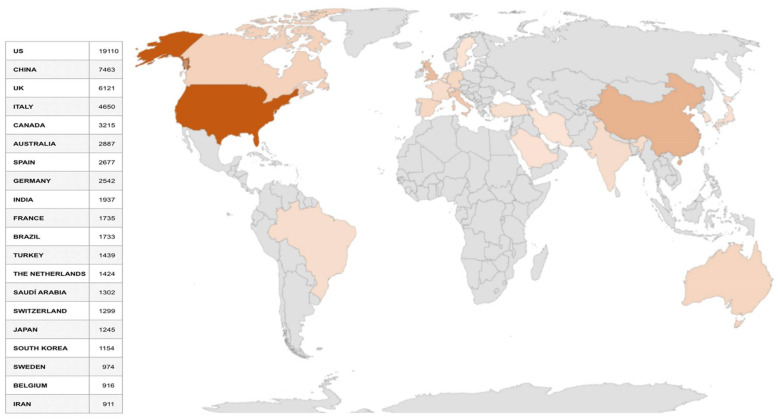
Countries with the highest scientific output on COVID-19.

In this analysis, a study has been carried out for each of the variables. Thus, firstly, the ranking of the 25 countries that have published the most on COVID-19 is shown (Figure 1). The USA and China are followed by the UK, Italy, Canada, Australia and Spain.

It can be seen that countries with a strong pressure of coronavirus in their territories, researchers have carried out a very important scientific production.

Table 6 shows the COVID-19 or Coronavirus and Health search terms for the countries, number of manuscripts and citations for each of them. The four most scientifically productive countries remain the same: USA, China, UK and Italy.

The European Union has been one of the most affected by the different waves of contagion and has defined some common strategies to face the pandemic, such as vaccines or the COVID-19 passport. Although it is understood that the best way to see scientific production is broken down, if all the scientific production of the European Union were to be combined, the number of publications would amount to 10,081 manuscripts, placing it in first place. It should be noted that since Brexit, the United Kingdom is no longer counted within the European Union. In the case of the United Kingdom, WoS breaks down the data into England with 3270 and Scotland with 444 papers.

Table 7 shows the data subject to the COVID-19 and ‘Health Communication’ study. Once again, the same four countries repeat in the ranking in the field of Health Communication, although in this case Spain is in fifth place. In the European Union as a whole, 56 manuscripts were published in this ranking of 25 countries.

### 3.5. Analysis of the Main Organisations and Number of Papers

#### COVID-19 Analysis and Health Communication

Focusing again on the object of study for COVID-19 and Health Commnication, Table 8 presents a ranking of the most influential international affiliations through reference universities together with indicators of registrations and the percentage that these represent in the total sum of articles analysed. In addition, following the classification of Sánchez-Núñez, de las Heras-Pedrosa and Peláez [28], two global university ranking indicators are added for the year 2021 QS World University Rankings and Academic Ranking of World Universities (ARWU) that allow measuring the relative position in which the most influential institutions are located.

**Figure 2:**
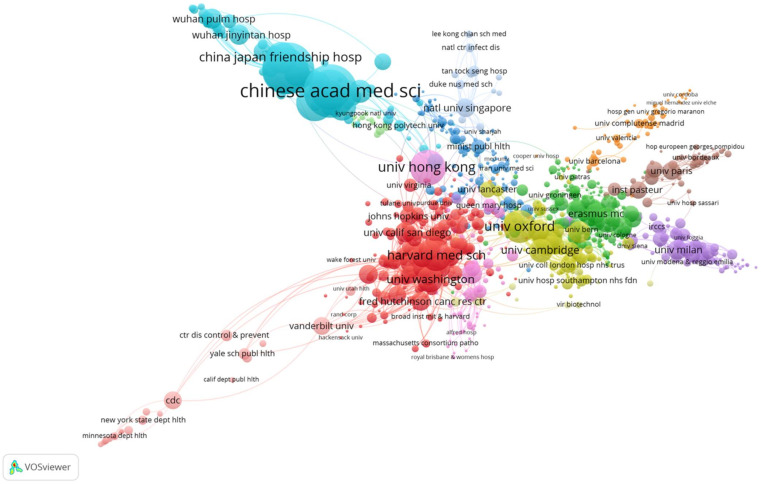
Most influential organisations in the body of publications on health communication and COVID-19.

Figure 2 allows us to distinguish the networks of the most influential academic institutions as a result of their citations. The university networks are grouped into clusters. An important cluster can be observed in Chinese institutions and another in United States universities. This is followed by the cluster of British universities. In the case of the European Union, the clusters are subdivided according to their importance in the universities of Italy, France and Spain, and unified in another cluster for the rest of Europe.

### 3.6. Main Funding Organisations

The Top 10 entities that have financially supported the most research for the publication of articles have been, in order from the highest number of funded registrations to the lowest: National Institutes of Health Nih USA; United States Department of Health Human Services; European Commission; National Science Foundation Nsf; Google Incorporated; Grants in Aid for Scientific Research Kakenhi; Japan Society for the Promotion of Science; Ministry of Education, Culture, Sports, Science and Technology Japan Mext; National Natural Science Foundation of China Nsfc; Swiss National Science Foundation Snsf.

### 3.7. Research Areas and Record Counts

From the five general thematic areas in which the different manuscripts registered in WoS are classified (Arts and Humanities, Life Sciences, Biomedicine, Physical Sciences, Social Sciences and Technology), other thematic subcategories are derived.

#### 3.7.1. Study for COVID-19 and Health

**Figure 3:**
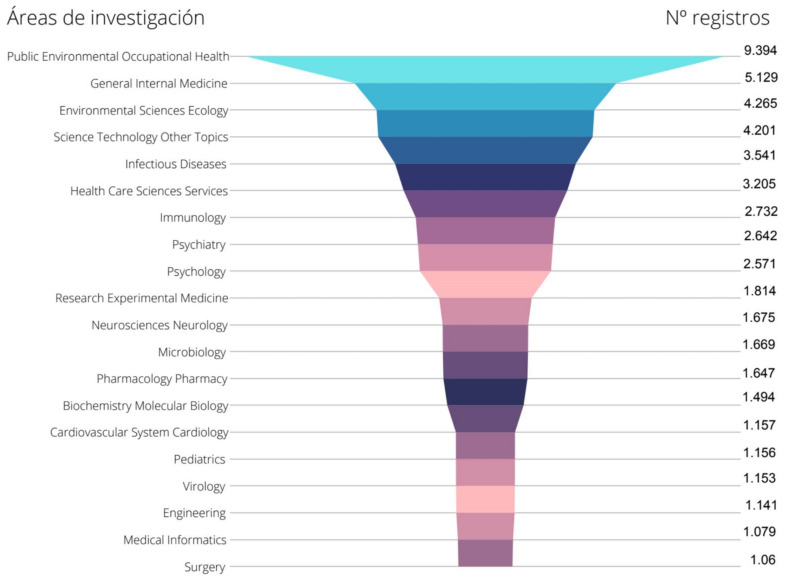
Research fields and records by category over the total number of analysed articles of COVID-19 and Health.

The main areas of research are reflected in Figure 3. Among those areas of knowledge that have more than 3000 papers are: ‘Public Environmental Occupational Health’ covers the largest number of records, followed by the fields related to: ‘General Internal Medicine’, ‘Environmental Sciences Ecology’, ‘Science Technology Other Topics’ ‘’and ‘Environmental Sciences Ecology’.

#### 3.7.2. COVID-19 Analysis and Health Communication

**Figure 4:**
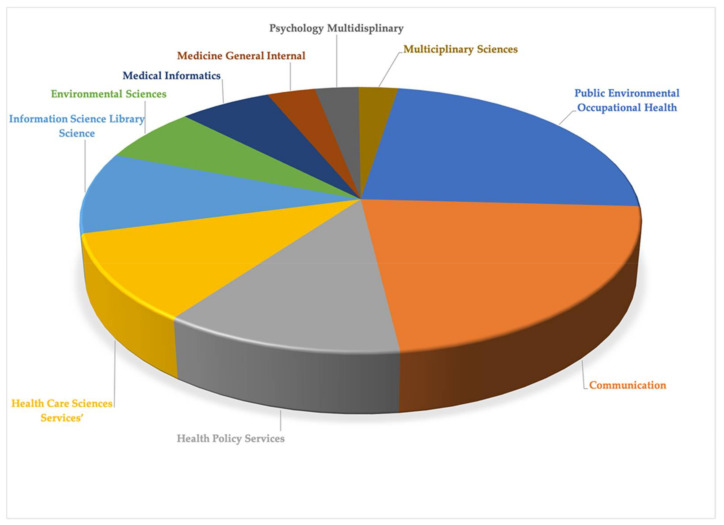
Research fields and records by category over the total number of analysed COVID-19 and health communication articles.

In the articles selected after searching for articles in which ‘Health Communication’ appeared next to the concept COVID-19 or Coronavirus’, the 10 research areas in which the highest number is assigned are: ‘Public Environmental Occupational Health’ (30.01% of the total); ‘Communication’ (28.57%); ‘Health Policy Services’ (15.41%); ‘Health Care Sciences Services’ (13.91%); ‘Information Science Library Science’ (12.78%); ‘Environmental Sciences’ (8.65%); ‘Medical Informatics’ (7.90%); ‘Medicine General Internal’ (4.14%); ‘Psychology Multidisciplinary’ (3.76%); ‘Multidisciplinary Sciences (3.39%) (Figure 4).

Comparing the data of the two cartographic maps (COVID-19 and Health) and (COVID-19 and Health Communication) (Figure 3 and Figure 4), it can be seen that the topics of ‘Public Environmental Occupational Health’; ‘Health Care Sciences Services’; ‘Environmental Sciences’; ‘Medicine General Internal’; and ‘Psychology’ are repeated.

### 3.8. Co-Occurrence Analysis

The most frequently repeated words in the analysed articles are represented visually. The graphs displayed after the analysis carried out with the VOSViewer tool also provide information on the terms that form fields or clusters of words that appear related in the same publication.

#### 3.8.1. COVID-19 Analysis

**Figure 5:**
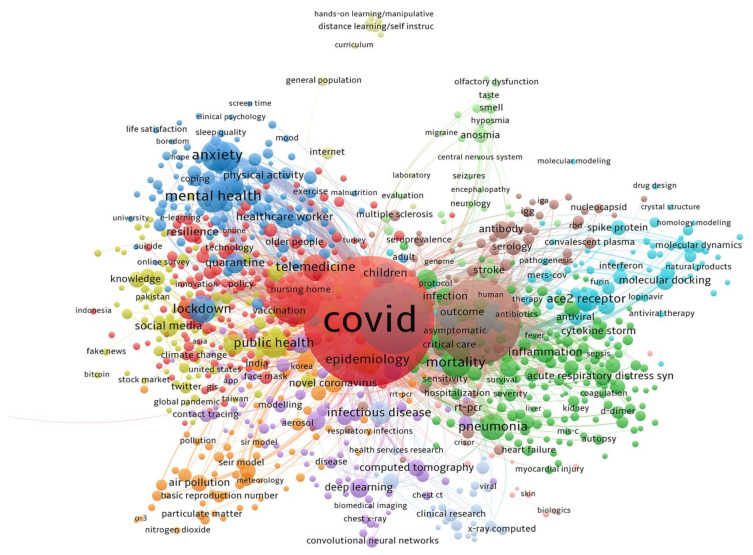
Map of co-occurrence after searching for the terms COVID-19 or coronavirus.

With a minimum number of occurrences of a keyword of one, following the same methodological process, from the most general to the most specific analysis, the map of co-words has been determined after searching in WoS for the terms COVID-19 or coronavirus. Thus, Figure 5 is obtained, in which, together with the predominant and most strongly related nuclei grouped under the nomenclature of COVID-19 and coronavirus, other outstanding semantic fields appear, such as those related to Mental Health, Public Health, Symptomatology and Treatments and Media, among other clusters of lesser relevance, but closely related to each other.

Figure 5 shows that the most important clusters—because they include the terms with the highest prevalence in the published articles and the strongest interrelation with other words or groups of words—are, in descending order, the following:

Cluster 1, with the word ‘covid’ heading this group, which shares a core with terms such as ‘pandemic’, ‘telemedicine’, ‘social distancy’ or ‘resilience’;

Cluster 8, with the term ‘sars coronavirus’ as predominant, together with ‘diagnostic’, ‘rt-pcr’ ‘antibody’ or ‘serology’;

Cluster 6, in which the term ‘coronavirus’ is followed by ‘receptor’, ‘hydroxy chloroquine’, ‘molecular docking’ or ‘spike protein’.

Of these three clusters, the strength of the relationship they maintain with other clusters and these clusters with each other stands out, such as:

Cluster 3, with related terms: ‘mental health’, ‘anxiety’, ‘depression’, ‘lockdown’, ‘stress’, ‘quarantine’, ‘psychological impact’ or ‘healthcare workers’;

Cluster 2, related to symptomatology: ‘mortality’, ‘pneumonia’, ‘inflammation’, ‘risk factor’, ‘pneumonia’, ‘prognosis’, ‘diabetes’, ‘pregnancy’, ‘infection’.

Cluster 4 is noteworthy for dealing specifically with aspects related to the field of study in question. In this cluster, ‘public health’ stands out above the terms of the group in which it is related, followed by the words ‘social media’, ‘vaccine’ and ‘vaccination’, ‘china’, ‘prevention’, ‘knowledge’, ‘attitude’, ‘survey’, ‘risk communication’, ‘misinformation’, ‘disinformation’ and ‘conspiracy theories’, ‘network analysis’, ‘infodemiology’ or ‘fake news’.

#### 3.8.2. COVID-19 Analysis and Health Communication

In terms of content, when analysing the keyword analysis by year with VOSviewer in those articles dealing with COVID-19 and Health Communication, there are similarities and differences in the predominant subject matter in one year or another, both in terms of the number of publications and the citations they have received.

The most recurrent terms are those repeated in the articles published in 2020 and 2021, especially those related to the names with which the pathology is designated and directly related: pandemic, coronavirus, COVID-19, SARS or Public Health. What has been also found in both years are those referring to preventive measures: Behavior and Planned Behavior. Others, such as Health Communication or Mass Communication, change from one year to another in the order of prevalence. Thus, in the year 2020, articles on the media were more widely disseminated than those on health communication, although health communication gained prominence in scientific publications on the subject under study.

**Figure 6:**
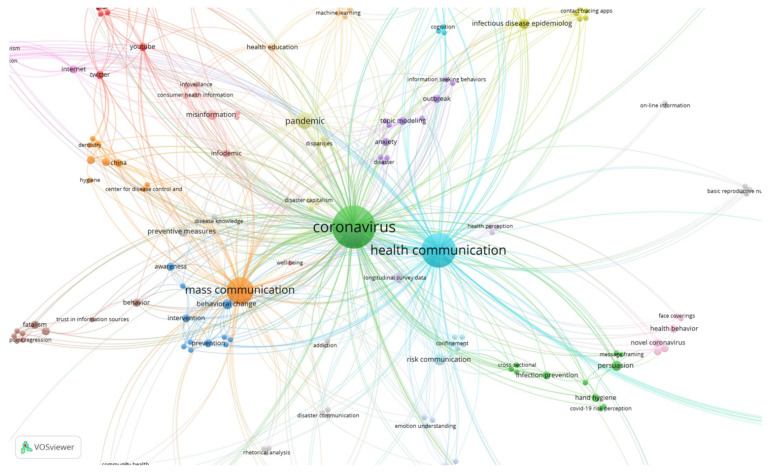
Map of keywords and their confluence in articles published in 2020.

**Figure 7:**
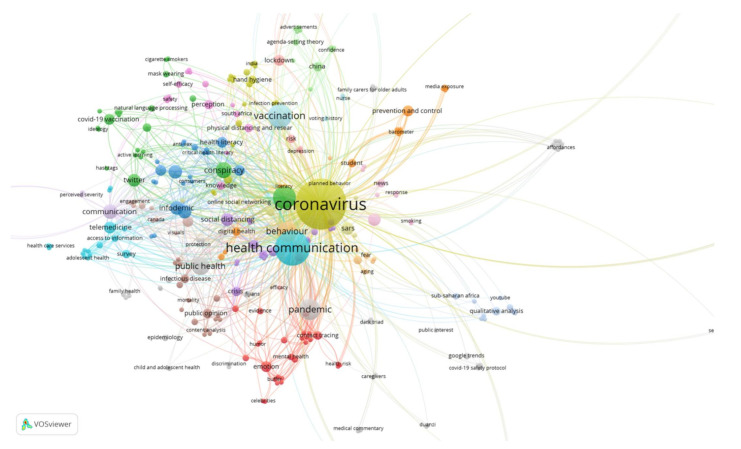
Map of keywords and their confluence in articles published in 2021.

Terms disappeared from the selected publications and others emerged over the months. In 2020, concepts related to Mental Health were more common: Anxiety, Awareness, Fatalism. In 2021, these were Vaccination, Risk Perception, Social Distancing, Health Promotion, or Telemedicine, which were not among the most prevalent keywords in the articles selected in 2020. From the terms Misinformation, Communication Crisis or Communication Risk in 2020, they changed in 2021 to the concepts of Infodemic, Conspiracy or Risk Perception, which denotes a difference in how information is received by society, depending on the time period (Figure 6 and Figure 7).

**Figure 8:**
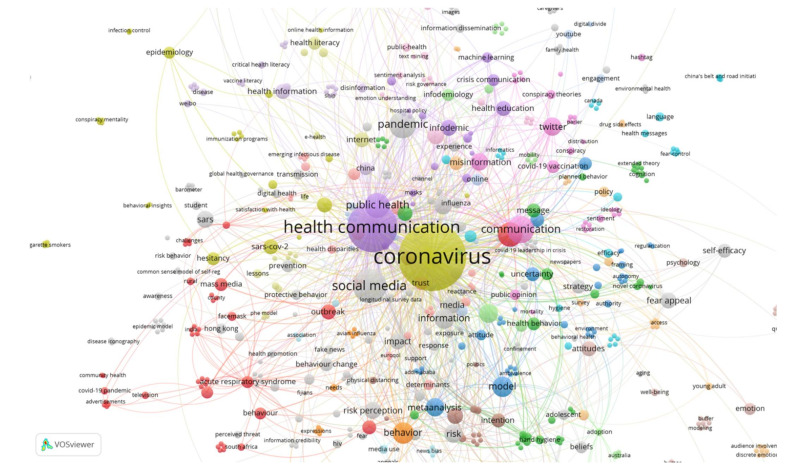
Co-occurrence map for the years 2020 and 2021.

The cluster analysis for the two years, jointly, is performed on 877 keywords highlighted by the authors. Thus, 44 different clusters are distinguished (Table A1) (Figure 8).

In this case, the main words at the top of the clusters that encompass terms with the highest prevalence in the published articles and that have a closer relationship with other terms are:

Cluster 4: ‘coronavirus’ is followed by ‘vaccination’, ‘internet’, ‘SARS-cov-2’, ‘epidemiology’, ‘hesitancy’, ‘health literacy’, ‘health information’;

Cluster 5: next to ‘health communication’ are ‘public health’, ‘crisis communication’, ‘infodemic’, ‘health education’, ‘readability’;

Cluster 20: ‘social media’, ‘information’, ‘fake news’, ‘beliefs’, ‘preventive behavior’, ‘behavior change’, ‘awareness’, ‘intervention’;

Cluster 9: ‘communication’, ‘misinformation’, ‘Twitter’, ‘COVID-19 vaccination’, ‘public opinion’, ‘conspiracy’, ‘conspiracy theories’, ‘sentiment’;

Cluster 32: ‘pandemic’, ‘ambulatory care’, ‘primary health care’, ‘social media influencer’;

Cluster 1: ‘health’, ‘outbreak’, ‘mass media’, ‘acute respiratory syndrome’, ‘lockdown’, ‘behavior’, ‘knowledge’;

Cluster 3: ‘social distance’, ‘news’, ‘uncertainty’.

The main clusters cited are closely related to each other and to other groups of terms, among whose most prominent keywords are: ‘fear appeal’, ‘risk perception’, ‘perception’, ‘behavior’, ‘attitudes’, ‘self-efficacy’, ‘risk perception’, ‘perception’, ‘behavior’, ‘attitudes’, ‘self-efficacy’, ‘self-efficacy’ and ‘knowledge’.

### 3.9. Analysis of the Most Cited Manuscripts

The analysis of the 25 most cited articles on coronavirus and health communication provides information on the topics that have had the greatest influence on the scientific community (Table 9).

The article entitled: ‘‘Considering emotion in COVID-19 vaccine communication: addressing vaccine hesitancy and fostering vaccine confidence’’ [57] is the one with the highest number of citations, 98. In this article, the authors present a battery of reasons why the United States population may be reluctant to be vaccinated against COVID-19. They propose, in turn, communication strategies based on the dissemination of scientific evidence of the efficacy of vaccines to promote greater confidence in them, as well as on the analysis of emotions, as an element to be taken into account in the construction of the message.

Two other articles have more than 50 citations during the years analysed:

- ‘‘The effects of social media use on preventive behaviors during infectious disease outbreak: the mediating role of self-relevant emotions and public risk perception’’ [58]. The relationship between what is communicated through social networks and the public’s perception of risk and preventive behaviors during infectious disease outbreaks is the topic developed by the authors of this article. In their findings, they highlight how two emotions, such as fear and anger, as well as the public’s perception of risk are positively related to social networks. The research also shows how the use of these communication channels can significantly increase preventive actions against the aforementioned pathologies;
- ‘Health communication through news media during the early stage of the COVID-19 outbreak in China: digital topic modeling approach’ [59]. With China as the setting, in this article the authors study the relevance of mass media in health communication during an early stage of the coronavirus. The analysis of the subject matter of the published news and their publication dynamics during this period provided findings such as the delay of media news reports in China with respect to the development of the pandemic.

**Figure 9:**
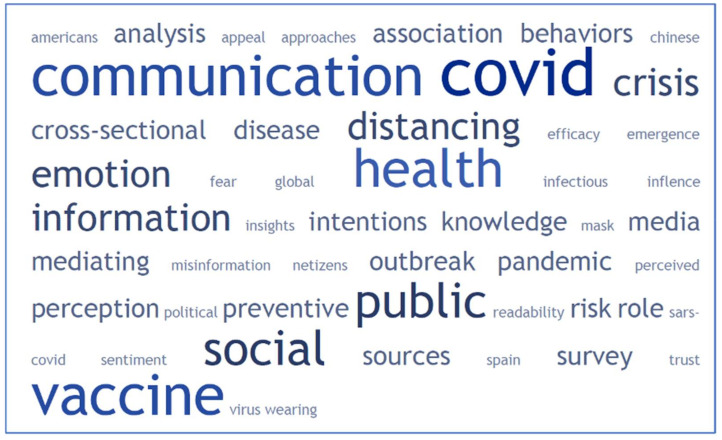
Predominant Word cloud in the scientific articles with the highest number of citations on COVID-19 and health communication.

In the word cloud, Figure 9, in addition to the terms ‘communication’, ‘covid’, ‘health, ‘public’, ‘social’ and ‘vaccine’, directly related to the subject of this work, other terms stand out that indicate aspects of what the scientific community is researching in this field: ‘social behaviors’, ‘crisis’, ‘emotions’, ‘social distance’, ‘information’, ‘behavior’, ‘media’, ‘perception’, ‘political communication’, ‘prevention’, ‘sources’, ‘social distance’ and ‘risk roles’, among others.

## 4. Discussion

This research presents the current status and trends of COVID-19 research and health communication. Although the scientific community has shown an unprecedented effort in generating a large number of studies to solve the problem, there is a need for a global characterisation in the different areas, given the existing multidisciplinarity [78].

Although there are several bibliometric studies on COVID-19 and Health [79,80], socio-economic [81], Communication [82] and Tourism [83] are identified as other additional factors. However, this would be one of the first bibliometric analyses addressing health communication at the time of COVID-19 by identifying in WoS the most productive authors, reference articles, universities, countries and research topics. As for the focus of study in the analysed works, a change of topic is observed in parallel to the evolution of the pandemic.

The results of the bibliometric analysis also indicate that there is a relative concentration of the most influential papers among a certain number of researchers, in contrast to the study in the field of health by ElHawary et al. [79]. In agreement with Torres Salinas [84], it is found that most of the articles are open access, which has led to a wide and rapid dissemination of contributions and, as the field continues to mature, numerous authors joining this line of research. It should also be noted that the number of citations is on the rise, demonstrating the current importance of the relationship under analysis.

The analysis of the geographical dispersion of the publications showed that the USA and China are the countries that contribute the highest number of papers, coinciding with other studies [79]. The results reveal the breadth of methodologies and disciplines used, even among the most prolific scholars, exemplifying the interdisciplinarity of health communication research.

### Limitations

COVID-19 has caused possibly the largest concentration of scientific resources, without precedent. Despite the existence of a multitude of resources, such as repositories and their important contribution, journals are the core of scientific production.

One of the debates at the beginning of this research was the use of the WoS or Scopus databases of bibliographic references and citations of periodicals. Both include prestigious journals and rigorous peer review, although it is true that normally the journals that are in WoS in its Social Sciences Citation Index (SSCI) and Science Citation Index Expanded (SCI-Expanded) databases are included in Scopus. Other WoS databases, such as Emerging Sources Citation Index, were not studied in the search for top-level references. For this reason, it was decided to use only the main scientific database, WoS.

On the other hand, the search term and the use of the field tag with the topic TS= ‘health communication’ should also be noted. Although other terms such as information, social media or public relations would have provided more results, the main health journals specialised in communication choose this denomination even for the title of their publication. This is the case of Health Communication or Journal of Health Communication, both of which are indexed in Journal Citation Reports (JCR) in Q2. Moreover, other health journals such as the International Journal of Environmental Research and Public Health are indexed in JCR in Q1 (social sciences) with a section called health communication. Hence, given that the main health journals determine the term ‘health communication’ as their main topic, this was chosen as the main topic for the selection of articles.

It was decided to carry out this research at a time when vaccines were already giving results and infections were at level 0, according to the European classification. However, it is true that a new variant of SARS-CoV-2, called Omicron, detected in November 2021, is causing a new wave of infections at the time of writing. The effects of the pandemic, according to the scientific director of Pfizer, Mikael Dolsten, will last until 2024 [85]. Therefore, the research on health communication and COVID-19 is not completed.

Finally, it should be noted that the results obtained also suggest future lines of research. It has been found that research on the impact of the crisis has focused mainly on the areas of ‘Public Environmental Occupational Health’, ‘Communication’ and ‘Health Policy Services’. Therefore, it would be interesting to address new areas of study to expand on the results obtained. For example, the impact of health communication in growing economies and in different target groups remains to be studied. The current analysis shows a concentration of contributions in a certain number of countries, so it would be necessary to broaden it and offer a global point of view.

## 5. Conclusions

Interest in scientific research in the field of health communication and COVID-19 is growing exponentially and the expected trend is that it will continue to increase. It should be noted that most of the publications that have faced the pandemic scenario have implemented open access policies to share their resources, which has contributed to the rapid diffusion of scientific information.

In general, there is also a significant difference between the articles published in 2020 and those disseminated the following year. The scientific literature is in line with the spread of the coronavirus worldwide and the communicative actions that accompany it. Terms such as fear, anxiety, mental health, awareness, resilience or lockdown in 2020 change in 2021 to behavior, vaccination, risk perception, social distance health promotion, or telemedicine. That said, it is true that some terms such as uncertainty are maintained over time, despite the fact that the role of health communication is to avoid precisely this feeling.

The information obtained from the analysis of authorship includes the references in the research with the greatest impact in the academic journals indexed in the WoS database analysed. The COVID-19 crisis was the first pandemic to be transmitted in real time. One of the great challenges has been to combat disinformation and fake news. It is therefore necessary for governments and health institutions to design clear communication strategies that are adapted to the different stages of the pandemic to avoid uncertainty, confusion or denial among the population. The lack of such strategies could lead to the failure of a correct public health policy.

## Figures and Tables

**Table 1:** Results of the searches carried out in the selection of archives for the bibliometric analysis.

	Date	Search	Filters			Publications
			Document Types	Languages	Indexes	
1	23/11/21	TS = ((COVID-19) OR (coronavirus))	Articles	English	- SSCI - SCI-Expanded	85,988
2	23/11/21	TS = ((COVID-19) OR (coronavirus) AND (health))	Articles	English	- SSCI - SCI-Expanded	52,416
3	23/11/21	TS = (((COVID-19) OR (coronavirus)) AND (‘health communication’))	Articles	English	- SSCI - SCI-Expanded	493 articles from which 262 are extracted, after applying the above-mentioned filters.

**Table 2:** Citation report (COVID-19/‘Health communication’).

Citation Report	
Total number of publications	262
Sum of citations	1505
Average citation per article	5.74
H-index	20

**Table 3:** Ranking of leading journals with publications on COVID-19 and Health.

Ranking	Journals	Papers	Journal Impact Report (JCR, 2020)	
			Science	Social Science
1	International Journal of Environmental Research and Public Health	2541	Q2	Q1
2	Plos One	1303	Q2	-
3	Frontiers in Public Health	869	Q2	Q1
4	Frontiers in Psychology	741	-	Q2
5	Scientific Reports	681	Q1	-
6	BMJ Open	582	Q2	-
7	Journal of Medical Internet Research	534	Q1	-
8	Frontiers in Psychiatry	466	Q2	Q2
9	International Journal of Infectious Diseases	408	Q2	-
10	BMC Public Health	396	Q2	-
11	Sustainability	383	-	Q2
12	Journal of Clinical Medicine	329	Q1	-
13	Vaccines	329	Q2	-
14	Nature Communications	312	Q1	-
15	Journal of Medical Virology	287	Q4	-
16	Science of the Total Environment	283	Q1	-
17	Frontiers in Medicine	275	Q1	-
18	Jama Network Open	268	Q1	-
19	Heatlhcare	264	Q3	Q2
20	Frontiers in Immunology	241	Q1	-

**Table 4:** Ranking of leading journals with articles on COVID-19 and health communication.

Journals	Articles	Citations	Journal Impact (JCR, 2020)		% of Total
			Science	Social Science	
Journal of Health Communication	34	93	-	Q2	11.28%
Health Communication	30	383	-	Q2	10.53%
International Journal of Environmental Research and Public Health (IJERPH)	24	202	Q2	Q1	7.89%
Journal of Medical Internet Research	20	197	Q1	-	7.52%
Frontiers in Public Health	13	25	Q2	Q1	4.13%
Frontiers in Psychology	10	64	-	Q2	3.01%
Healthcare	6	26	Q3	Q2	2.26%
Multilingua—Journal of Cross-Cultural and Interlanguage Communication	5	34	-	Q2	1.88%
Social Science & Medicine	5	83	Q1	Q1	1.88%
BMJ Open	4	14	Q2	-	1.50%
PLoS ONE	4	34	Q2	-	1.50%
Vaccines	4	6	Q2	-	1.50%
Asia-Pacific Journal of Public Health	3	0	Q4	Q4	1.13%
Epidemiology and Infection	3	5	Q3	-	1.13%
JMIR Public Health and Surveillance	3	77	Q2	Q1	1.13%
Media International Australia	3	20	-	Q4	1.13%
Patient Education and Counseling	3	14	Q2	Q1	1.13%
PEERJ	3	15	Q2	-	1.13%
Vaccine	3	15	Q3	-	1.13%
American Journal of Tropical Medicine and Hygiene	3	10	Q2	-	1.13%

**Table 5:** Scientific production by authors.

Author	Articles	Journal
Latkin, Carl A.	- Coverage of Health Information by Different Sources in Communities: Implication for COVID-19 Epidemic Response (2020) [33].	International Journal of Environmental Research and Public Health
	- An Assessment of the Rapid Decline of Trust in US Sources of Public Information about COVID-19 (2020) [34].	Journal of Health Communication
	- Feasibility of Intersectoral Collaboration in Epidemic Preparedness and Response at Grassroots Levels in the Threat of COVID-19 Pandemic in Vietnam (2020) [35]	Frontiers in public health
	- Knowledge and awareness of COVID-19 among Indonesian migrant workers in the Greater China Region. (2021) [36]	Public health
Bann, Carla M.	- Predictors of willingness to get a COVID-19 vaccine in the U.S. (2021) [37]	BMC Infectious Diseases
	- Mental Models of Infectious Diseases and Public Understanding of COVID-19 Prevention. (2020) [38]	Health Communication
	- Gaps in Knowledge About COVID-19 Among US Residents Early in the Outbreak. (2021) [39]	Public health reports
Barello, Serena	- Critical observations on and suggested ways forward for healthcare communication during COVID-19: pEACH position paper. (2021) [40]	Patient education and counseling
	- ‘#I-Am-Engaged’: Conceptualization and First Implementation of a Multi-Actor Participatory, Co-designed Social Media Campaign to Raise Italians Citizens’ Engagement in Preventing the Spread of COVID-19 Virus. (2020) [41]	Frontiers in psychology
	- Effects of the COVID-19 Emergency and National Lockdown on Italian Citizens’ Economic Concerns, Government Trust, and Health Engagement: Evidence from a Two-Wave Panel Study. (2021) [42].	The Milbank quarterly
Bleakley, Amy	- Psychosocial Determinants of COVID-19 Vaccination Intention Among White, Black, and Hispanic Adults in the US. (2021) [43]	Annals of behavioral medicine: a publication the Society of Behavioral Medicine
	- Uncertainty Management and Curve Flattening Behaviors in the Wake of COVID-19’s First Wave. (2021) [44]	Health Communication
	- Ideological Health Spirals: An Integrated Political and Health Communication Approach to COVID Interventions. (2020) [45]	International Journal of Communication
De las Heras-Pedrosa, Carlos	- Resilience and Anti-Stress during COVID-19 Isolation in Spain: An Analysis through Audiovisual Spots. (2020) [46]	International Journal of Environmental Research and Public Health
	- Sentiment Analysis and Emotion Understanding during the COVID-19 Pandemic in Spain and Its Impact on Digital Ecosystems. (2020) [3]	International Journal of Environmental Research and Public Health
	- Exploring the Social Media on the Communication Professionals in Public Health. Spanish Official Medical Colleges Case Study. (2020) [19]	International Journal of Environmental Research and Public Health
Graffigna, Guendalina	- Critical observations on and suggested ways forward for healthcare communication during COVID-19: pEACH position paper. (2021) [40]	Patient education and counseling
	- ‘#I-Am-Engaged’: Conceptualization and First Implementation of a Multi-Actor Participatory, Co-designed Social Media Campaign to Raise Italians Citizens’ Engagement in Preventing the Spread of COVID-19 Virus. (2020) [41]	Frontiers in psychology
	- Effects of the COVID-19 Emergency and National Lockdown on Italian Citizens’ Economic Concerns, Government Trust, and Health Engagement: Evidence from a Two-Wave Panel Study. (2021) [42].	The Milbank Quarterly
Jambrino Maldonado, Carmen and Iglesias-Sánchez, Patricia P.	- Resilience and Anti-Stress during COVID-19 Isolation in Spain: An Analysis through Audiovisual Spots. (2020) [46]	International Journal of Environmental Research and Public Health
	- Exploring WHO Communication during the COVID 19 Pandemic through the WHO Website Based on W3C Guidelines: Accessible for All? (2020) [47]	International Journal of Environmental Research and Public Health
	- The Contagion of Sentiments during the COVID-19 Pandemic Crisis: The Case of Isolation in Spain (2020) [4]	International Journal of Environmental Research and Public Health
Hwang, Juwon	- Polarization over vaccination: ideological differences in Twitter expresion about COVID-19 vaccine favor ability and specific hesitancy concerns (2021) [48]	Social Media + Society
	- COVID-19 Vaccination Attitudes and Intention: Message Framing and the Moderating Role of Perceived Vaccine Benefits. (2021) [49]	Journal of Health Communication
	- Health Information Sources and the Influenza Vaccination: The Mediating Roles of Perceived Vaccine Efficacy and Safety (2020) [50]	Journal of Health Communication
McCormack, Lauren A.	- Gaps in Knowledge About COVID-19 Among US Residents Early in the Outbreak. (2021) [39]	Public Health Reports
	- Predictors of willingness to get a COVID-19 vaccine in the U.S. (2021) [37]	BMC Infectious Deseases
	- Mental Models of Infectious Diseases and Public Understanding of COVID-19 Prevention. (2020) [38]	Health Communication
Parker, Ruth M.	- Face Masks: Their History and the Values They Communicate (2021) [51]	Journal of Health Communication
	- COVID-19: An Urgent Call for Coordinated, Trusted Sources to Tell Everyone What They Need to Know and Do. (2020) [52]	NAM Perspectives.
	- Building Vaccine Literacy in a Pandemic: How One Team of Public Health Students Is Responding. (2020) [53]	Journal of Health Communication
Resniscow, Ken	- Relationship Between Coronavirus-Related eHealth Literacy and COVID-19 Knowledge, Attitudes, and Practices among US Adults: Web-Based Survey Study (2021) [54]	Journal of Medical Internet Research
	- Novel Predictors of COVID-19 Protective Behaviors Among US Adults: Cross-sectional Survey. (2021) [55]	Journal of Medical Internet Research
	- Perspectives on Oncology-Specific Language During the Coronavirus Disease 2019 Pandemic: A Qualitative Study. (2020) [56]	JAMA Oncology

**Table 6:** Countries, number of articles and citations on COVID-19 and health.

COVID-19 and Health			
	Country	Documents	Citations
1	USA	9428	400,435
2	China	4889	428,240
3	England	3270	153,432
4	Italy	3208	115,542
5	Germany	1680	90,715
6	Spain	1530	56,909
7	Canada	1484	61,020
8	France	1413	69,086
9	Australia	1379	62,090
10	India	1372	31,308
11	Netherlands	829	51,219
12	Brazil	804	26,449
13	Switzerland	750	38,179
14	Turkey	635	15,706
15	Saudi Arabia	630	16,675
16	Japan	627	23,375
17	South Korea	602	23,588
18	Belgium	578	25,019
19	Iran	543	14,010
20	Sweden	511	19,266
21	Singapore	451	25,625
22	Scotland	444	25,875
23	Israel	396	11,905
24	Denmark	332	19,599
25	Pakistan	331	9117

**Table 7:** Countries, number of articles and citations on COVID-19 and ‘Health Communication’.

COVID-19 and Health Communication			
	Country	Documents	Citations
1	USA	130	858
2	China	28	242
3	England	24	151
4	Italy	12	91
5	Spain	12	102
6	Australia	11	138
7	Germany	11	48
8	South Korea	11	113
9	Canada	10	43
10	Switzerland	9	34
11	Denmark	6	9
12	Japan	5	29
13	Netherlands	5	7
14	Israel	4	17
15	Nigeria	4	11
16	Portugal	4	19
17	Singapore	4	78
18	Austria	3	4
19	Belgium	3	5
20	Colombia	3	21
21	France	3	17
22	India	3	7
23	Malaysia	3	1
24	South Africa	3	0
25	Vietnam	3	58

**Table 8:** Most reputed international universities.

Clasification	Organisation	Articles	% Total	QS 2021	ARWU 2021
1.	University of North Carolina	10	3.759	295	801–900
2.	University of London	8	3.008	114	201–300
3.	University or North Carolina Chapel Hill	8	3.008	95	29
4.	Johns Hopkins University	7	2.632	25	16
5.	Pennsylvania Commonweallth System of Higher Education PSCHE	7	2.632	-	-
6.	University of California System	7	2.632	-	14
7.	Harvard University	6	2.256	3	1
8.	Johns Hopkins Bloomberg School of Public Health	6	2.256	-	-
9.	State University of New York Science System	6	2.256	373	601–700
10.	University of Michigan	6	2.256	21	26
11.	University of Michigan Health System	6	2.256	-	-
12.	Emory University	5	1.880	158	101–150
13.	New York University	5	1.880	35	27
14.	Pennsylvania State University	5	1.880	16	-
15.	University of Hong Kong	5	1.880	22	101–150
16.	University of Pennsylvania	5	1.880	16	15
17.	University System of Georgia	5	1.880	501–510	101–150
18.	Chinese University of Hong Kong	4	1.504	43	101–150
19.	Imperial College London	4	1.504	8	25
20.	London School of Hygiene Tropical Medicine	4	1.504	-	201–300
21.	National Institutes of Health NIH USA	4	1.504	-	-
22.	Pennsylvania State University Park	4	1.504	101	101–150
23.	University of Copenhagen	4	1.504	76	30
24.	University of Málaga	4	1.504	-	701–800
25.	University of Wisconsin System	4	1.504	65	31

**Table 9:** Articles with the highest citation index on COVID-19 and health communication.

Manuscript Title	Authors	Journals	Year	Citation (WoS)
Considering emotion in COVID-19 vaccine communication: addressing vaccine hesitancy and fostering vaccine confidence	Chou, W.Y.S.; Budenz, A. [57]	Health Communication	2020	98
The effects of social media use on preventive behaviours during infectious disease outbreaks: the mediating role of self-relevant emotions and public risk perception	Oh, S.H.; Lee, S.Y.; Han, C. [58]	Health Communication	2021	87
Health communication through news media during the early stage of the COVID-19 outbreak in China: digital topic modeling approach	Liu, Q.; Zheng, Z.; Zheng, J.; Chen, Q.Y.; Liu, G.; Chen, S.H; Chu, B.J.; Zhu, H.Y.; Akinwunmi, B.; Huang, J.; Zhang, C.J.P.; Ming, W.K. [59]	Journal of Medical Internet Research	2020	57
The emergence of COVID-19 in the US: a public health and political communication crisis	Gollust, S.E.; Nagler, R.H.; Fowler, E.F. [60]	Journal of Health Politics Policy and Law	2020	42
A national survey assessing SARS-COVID-2 vaccination intentions: implications for future public health communication efforts	Head, K.J.; Kasting, M.L.; Sturm, L.A.; Hastsock, J.A.; Zimet, G.D. [61]	Science Communication	2020	40
Association between public knowledge about COVID-19, trust in information sources, and adherence to social distancing: cross-sectional survey	Fridman, I.; Lucas, N.; Henke, D.; Zigler, C.K. [62]	JMIR Public Health and Surveillance	2020	33
How fear appeal approaches in COVID-19 health communication may be harming the global community	Stolow, J.A.; Moses, L.M.; Lederer, A.M.; Carter, R. [63]	Health Education & Behavior	2020	30
Social distancing and stigma: association between compliance with behavioral recommendatios, risk perception, and stigmatizing attitudes during the COVID-19 outbreak	Tomczyk, S.; Rahm, M.; Schmidt, S. [64]	Frontiers in Psychology	2020	30
Association of COVID-19 misinformation with face mask wearing and social distancing in a nationally representative US sample	Hornik, R.; Kikut, A.; Jesch, E.; Woko, C.; Siegel, I.; Kim, K. [65]	Health Communication	2021	29
Influence of social media platforms on public health protection against the COVID-19 pandemic via the mediating effects of public health awareness and behavioral changes: integrated model	Al-Dmour, H.; Masa’deh, R.; Salman, A.; Abuhashesh, M.; Al-Dmour, R. [66]	Journal of Medical Internet Research	2020	29
Sentiment analysis and emotion understanding during the COVID-19 pandemic in spain and its impact on digital ecosystems	De las Heras-Pedrosa, C.; Sanchez-Nunez, P.; Pelaez, J.I. [3]	International Journal of Environmental Research and Public Health (IJERPH)	2020	24
Protection motivation and the COVID-19 virus	Kowalski, R.M.; Black, K.J. [67]	Health Communication	2021	24
Can a COVID-19 vaccine live up to americans expections? A conjoint analysis of how vaccine characteristics inflence vaccination intentions	Motta, M. [68]	Social Science & Medicine	2021	22
Social media use, ehealth literacy, disease knowledge, and preventive behaviors in the COVID-19 pandemic cross-sectional study on Chinese netizens	Li, X.J.; Liu, Q.L. [69]	Journal of Medical Internet Research	2020	20
Fatalism in the context of COVID-19; perceiving coronavirus as a death sentence predicts reluctance to perform recommended preventive behaviors	Jiménez, T.; Restar, A.; Helm, P.J.; Cross, R.I.; Barath, D.; Arndt, J. [70]	SSM-Population Helth	2020	19
The Contagion of Sentiments during the COVID-19 Pandemic Crisis: The Case of Isolation in Spain	Iglesias-Sánchez, P.; Vaccaro Witt, G.; Cabrera, F.E.; Jambrino-Maldonado, C. [4]	International Journal of Environmental Research and Public Health (IJERPH)	2020	17
Public health communication in time of crisis: readability of on-line COVID-19 information	Basch, C.H.; Mohlman, J.; Hillyer, G.C.; Garcia, P. [71]	Disaster Medicine and Public Health Preparedness	2020	16
Coverage of the COVID-19 in the online versions of highly circulated US daily newspaper	Basch, C.H.; Kecojevic, A.; Wagner, V.H. [72]	Journal of Community Health	2020	13
(Mis)communicating about COVID-19: insights from health and crisis communication	Noar, S.M.; Austin, I. [73]	Health Communication	2020	11
How the health rumor misleads people’s perception in a public health emergency: lessons from a purchase craze during the COVID-19 outbreak in China	Zhang, L.W.; Chen, K.L.; Jiang, H.; Zhao, J. [74]	International Journal of Environmental Research and Public Health (IJERPH)	2020	8
Staying connected during COVID-19: the social and communicative role of an ethnic online community of Chinese international students in South Korea	Jang, I.C.; Choi, L.J. [75]	Multilingua-journal of Cross-cultural and Interlang. Com.	2020	7
Exploring WHO Communication during the COVID 19 Pandemic through the WHO Website Based on W3C Guidelines: Accessible for All?	Fernández-Díaz, E.; Iglesias-Sánchez, P.; Jambrino-Maldonado, C. [47]	International Journal of Environmental Research and Public Health (IJERPH)	2020	7
Public health messages about COVID-19 prevention in multilingual Taiwan	Chen, C.M. [76]	Multilingua-journal of Cross-cultural and Interlang. Com.	2020	6
Health information sources and the influenza vaccination; the mediating roles of perceived vaccine efficacy and safety	Hwang, J. [50]	Journal of Health Communication	2020	5
Examining persuasive message type to encourage staying at home during the COVID-19 pandemic and social lockdown: a randomized controlled study in Japan	Okuhara, T.; Okada, H.; Kiuchi, T. [77]	Patient Education and Counseling	2020	5

## Appendix A

**Table A1:** Cluster analysis. List of terms.

Cluster.	List of Terms
1	acute respiratory syndrome; adherence; advertisements; agenda-setting theory; behaviour; challenges; chinese; community health; community partnership; county; COVID-19 pandemic; disclosure; discourse;; facemask; handwashing; health; health advertising; health information seeking; knowledge; mass media; outbreak; pandemic influenza; participation; personal protective measures; physical distancing and research; recruitment; rural; school education; social behavioral changes; social capital; social determinants of health (mesh); social distancing behaviour; stay at home directive; television; work.
2	anti-vax; arousal; attitudinal inoculation; augmented reality; befefits; cognition; donor; extended theory; fear appeal message; fit indexes; hand hygiene; health behavior; human-computer interactions; infection prevention; inoculation; message; message framing; moralization; need; novel coronavirus; pandemic response; persuation; planned behavior; position; protection motivation; protection motivation theory; recall; resistance; scripts; self-focused attention; skills; social lockdown; survey research; transportation; vaccine hesitancy; vaccine uptake; willingness.
3	addis-ababa; ambivalence; attitude; authority; autonomy; behavioral response; breast-cancer; center for disease control; certainty; COVID-19 regulations; e-government; emotional appraisal; environment; framing; government; government Weibo; health-related behaviors; historical research; integration; media advocacy; metaanalysis; model; morality; news; patient; perceived belief; preferences; prosocial behavior; public health agencies; regularization; social distancing; threat; transparency; trust in information sources; uncertainty; will
4	attacks; behavioral insights; cigarette smokers; conspiracy mentality; coronavirus; epidemiology; health policy; hesitancy; immunization programs; infection control; injuries; mask wearing; media diet; medical-care; news-finds-me; online health information; online social endorsement; online social networking; organizasiontal development; rapid tests; sars-cov-2; satisfaction with health; satisfaction with healthcare; social factors; substance use disorder; threat perception; vaccination; vaccination coverage; vaccination hesitancy; vaccination refusal; vaccine trust; victims.
5	actionability; consumers; crisis communication; cross-cultural communication; deep learning; emergency; ethic; eye-tracking; face coverings; family-centered care; global health; health communication; health education; health systems; hospital policy; infodemic; information design; machine learning; masks; mathematical learning; natural language processing; patient education materials; personal protecting equipment; predict; public health; public perceptions; readability; scene; state health department; tool; understandability; visual attention
6	bilingual communication; citizen participation; COVID-19 crisis communication; crisis management; decision-making; departments; ebola; efficacy; english as a global language; epidemic; event representation; fear-control; freedom; gain; health messages; hygiene; informatics; language; level; multilingualism; organizations use; preparedness; psychological reactance; representations; resilience; self; social media use; vulnerability; warning labels
7	avian influenza; behavior; child and adolescent health; children; collectivism; computational social science; culture; decade; expressions; general-population; grounded theory; health belief model; hpv vaccination; individualism; information seeking behaviors; los-framed messages; mixed methods research; needs; palliative care; physician; principles; public health research; qualitative method; relative persuasiveness: rhethorical analysis; rural health; scholarly publishing; science communication; smoking-cessation; survelillance; topic models
8	affordable care act; age; anxiety; app; contact tracing; coverage; cross-sectional studies; fox news; framework; health behavior change; health beliefs; information-technology; intention; media fragmentation; mobile; physical distancing; political polarization; politics; privacy; reasoned action; reinforcing media spirals; security; social identity theory; social norms; technology acceptance; theory of planned behaviour; unified theory; user acceptance; utaut1; utaut2
9	active learning; communication; conspriacy; conspiracy theories; content analysis; COVID-19 vaccination; distribution; echo chamber; expression; hashtag; hpv vaccine; ideology; image repair; malaysian government; misinformation; mistrust in science; mortality; online discussions; parler; predictors; public opinion; public-policy; random forest; restoration; sentiment; supervised learning; time; twitter; unsupervised learning
10	care; complementary medicine; cultural competence; definition; determinants; disparities; emerging infectious disease; euroqol; evidence-based health messaging; flu vaccine; health disparities; health-risk; information use; interview; literacy; medical-students; outcomes; pandemic health communication; public health communication; public understanding of science; refugees; sex; trust; vaccine acceptance; vaccine willingness
11	adoption; consumer health information; contact-tracing app; COVID-19 intervention; digital media; disclosure intention; e-commerce; fsqca; health risk; human behavior; infectious disease; infectious disease outbreaks; infodemiology; infoveillance; mobility; moderating role; multimedia; perception; privacy risk; public health surveillance; risk-risk tradeoff; search; shelter-in-place; smartphones; surgery; variance
12	activists; breast; cancer; citizens; criticism; digital divide; digital literacy; engagement; epidemic preparedness; grassroots level; health information Exchange; health media; health professionals; improving influenza vaccination; instagram; intersectorial collaboration; networks; online; optimize; preventive measures; public crisis; system; taxonomy; text; topic modeling; war; youtube
13	communication campaign; community engagement; constructivism; consumer sentiment; e-health; education; ehealth; health engagement; health literacy; health-care; internet; lessons; life; patient education; patient engagement; patient health engagement, phe model; phe scale; prevention and control; quality; renal transplantation; social-epidemiological dimensions; teaching modes; vaccinarsi network; web; web-based information; website
14	argumentation theory; coronavirus infections; critical health literacy; critical thinking; dentistry; disease; disinformation; family cares for older adults; governance; health information; health information management; health system; infectious disease epidemiology; inforamtion appraisal; leadership; online health; oral health; sars virus; ship; sustainable ageing society; testing; tweet; vaccine literacy; weibo; world-wide-web
15	association; community; disaster; disaster communication; disease knowledge; ehealth literacy; emergency linguistics; english-centric multilingualism; intercultural dialogue in sociolinguistics; language brokering; laguage challenges of COVID-19; media use; mental-health literacy; multilingual crisis communication; new media; news bias; online community building; polarization; public health management; selective exposure; september-11; social representations; stress; symptoms; terrorist attacks; translation
16	access; adolescent health; audience involvement; bereavement conversations; celebrities; communication with masks; death; difference-in-differences; discrete emotions; disease names; drug side effects; health care seeking behavior; health education and promotion; health services; information seeking; media effects; parasocial interaction; policy; prevention strategies; primary care; steve jobs implications; survey; teenager; telemedicine; vaccine; young adult
17	attitudes; buffer; climate communication; communication infraestructure effective; emotion; emotion regulation; emotion exhaustion; environmental education; evidence; humor; information processing; modeling; multilevel approach; outreach; perspective, power; psychology; public communication; public engagement; risk information-seeking; social psychology; spillover; storylines; storytelling; well-being
18	addiction; anger; child development; cognitive appraisal; crisis; emotion understanding; for-disease-control; health misinformation; health perception; health rumor; health threats; judgment; live; opinion mining; perceived risk; public health emergency; public-heatlh; qualitative analysis; risk communication-research; risk governance; rumor; sentiment analysis; sub-national health workers; text mining; text mining analysis
19	belief determinants; caregivers; communicable diseases; environmental health; experience; family; family health; family reactions; icu; improve communication; information dissemination; intensive care units; newspapers, older people; people; physical-activity; professional; psychological distress; reasoned action approach; safety; school nurse education; school nurse knowledge; self-efficacy; social isolation; stay at home orders
20	awareness; behaviour change; campaign; epidemic model; family communication; information; intergenerational communication; internet-based data; intervention; jordan; longitudinal survey data; media audiences; media exposure; middle-aged parents; news media; patterns; programs; public health behavior; public health protection; response; self-determination theory; social media; social media platforms; stability; support
21	accuracy; aging; antisocial personality; boldness; conscientiousness; costs; dark triad; dirty dozen; extroversion; fear appeal; gender-differences; healthy behaviors; individual differences; machiavellianism; narcissism; personality; psychopathy; public health practice; quarantine; sexual-behavior; strategy; traits; triad
22	acceptability; adolescent; appeals; behavioral health; behavioral science; cessation; depresion; diagnosis; extended parallel process model; fear; framing effects; health psychology; optimistic bias; prevalence; quit attempts; risk perception; smoking; tobacco; unplanned school closure; unrealistic optimism; validity
23	acceptance; audience segmentation; channel; confidence; conspiracy beliefs; dimensions; exposure; hiv/aids; impact; internet usage; mass-media compaigns; norms; on-line information; perceived salience of information; prism; protection; psychological predictors; reactance; science; seeking; the COVID-19 knowledge; voting history
24	COVID-19 emergency responses; dual-gendered leadership; epistemic beliefs; herd-immunity; human-papillonavirus vaccination; interpersonal discussion; justification by authority; media coverage; medicine; men prime minister and premiers; nurse; prosocial values; provider recommendation; public health intervention; reflections; scientific-political communication; vaccination intention; women; women chief medical officers
25	cross-sectional; discrimination; disease iconography; disease prevention; fatalism; health promotion; hiv; hong kong; labor worker; latent class analysis; mental-illness; migrant health; perceived threat; politics of care; preventive behavior; psychological impact; residents; socio-cultural factors; stigma; toxic masculinity
26	app icon; auditory signals; belief model; comprehension; concreteness; design; exemplification; fight; identification; interface; medical icon; narratives, perceived quality; ranking test; semantic distance; usability; user performance; visual-search; zika
27	barometer; barrier gestures; common sense model of self-regulation; compliance; digital health; global health governance; governmental recommendation predictors; health-risk behaviors; observation; prevention; protective behavior; risk behavior; risk representations; student; technology public-private partnerships; transmision; university student
28	affordances; copyright gatekeepers; copyright law; fair-use; harm reduction; individuals; live music; mental health; music therapy; online takedown; pain; pwud; risk avoidance; sharing; speech; stakeholder; technology; therapeutic artifact
29	confinement; corporate ads; COVID-19 leadership in crisis; crisis and emergency communication; economic epidemiology; emergency risk communication; frame; framing theory; influenza; isolation; management; media; obesity; public-relations; risk communication; scott morrison; vaccine information
30	adults; belief; COVID-19 mortality, COVID-19 prevalence; health information sources; human-papillomavirus; influenza vaccination; medical professionals; national-survey; perceived vaccine efficacy; perceived vaccine safety; probability; risk; scoring rules; subjective beliefs
31	breakpoint regression; cov-2; fatalities; google trends; growth curve; infection; insights; public interest; sars; social distancing effectiveness; spread; trends
32	ambulatory care; disaster capitalism; influence; medica commentary; pandemic; primary care physicians; primary health care; qualitative research telemedicine; social media influencer; traditional chinese medicine; zhihu
33	buzzsumo; fake news; health crisis; information credibility; mass communication; negative emotions; psychological response; self protective behaviors
34	ableism; access to information; activism; deaf and hard of hearing; disability media studies; lawsuits; persons living with disabilities; social model of disability
35	cross-linguistic chinese-mongolian intertextuality; indigenous population in taiwan; mongolian verbal art; mongols in china; multilingual public health; social actor inclusion
36	college-students; hooking; sexual health; tinder; uncertainty reduction theory
37	dynamics; exponential growth bias; graphical communication; nudges; who safety measures
38	COVID-19 safety protocol; googi; kusaal; mabia languages; musical health communication
39	basic reproductive number; coronavirus infections, epidemiology; disease transmission, infectious; prevention & control; epidemiological monitoring
40	hotel image; hotel safety; hotel selection; social exchange theory
41	COVID-19 risk perception; hadwashing practices; nigeria
42	deontology; home confinement epidemics; utilitarianism ethics
43	communication ecology; perceived severity; perceived susceptibility
44	images; reliability; visuals
